# Hydrogel-Based Strategies for Intervertebral Disc Regeneration: Advances, Challenges and Clinical Prospects

**DOI:** 10.3390/gels10010062

**Published:** 2024-01-15

**Authors:** Shivam U. Desai, Sai Sadhananth Srinivasan, Sangamesh Gurappa Kumbar, Isaac L. Moss

**Affiliations:** 1 Department of Orthopedic Surgery, Central Michigan University, College of Medicine, Saginaw, MI 48602, USA; 2 Department of Orthopedic Surgery, University of Connecticut, Storrs, CT 06269, USA

**Keywords:** intervertebral disc degeneration, hydrogels, regeneration

## Abstract

Millions of people worldwide suffer from low back pain and disability associated with intervertebral disc (IVD) degeneration. IVD degeneration is highly correlated with aging, as the nucleus pulposus (NP) dehydrates and the annulus fibrosus (AF) fissures form, which often results in intervertebral disc herniation or disc space collapse and related clinical symptoms. Currently available options for treating intervertebral disc degeneration are symptoms control with therapy modalities, and/or medication, and/or surgical resection of the IVD with or without spinal fusion. As such, there is an urgent clinical demand for more effective disease-modifying treatments for this ubiquitous disorder, rather than the current paradigms focused only on symptom control. Hydrogels are unique biomaterials that have a variety of distinctive qualities, including (but not limited to) biocompatibility, highly adjustable mechanical characteristics, and most importantly, the capacity to absorb and retain water in a manner like that of native human nucleus pulposus tissue. In recent years, various hydrogels have been investigated in vitro and in vivo for the repair of intervertebral discs, some of which are ready for clinical testing. In this review, we summarize the latest findings and developments in the application of hydrogel technology for the repair and regeneration of intervertebral discs.

## 1. Introduction

As one of the leading causes of disability, lower back pain has, and continues to be, a leading cause of morbidity and hospitalizations across the globe [1]. The United States spends around $100 billion to $200 billion per year to address the economic burden of lower back pain, two thirds of which is caused by decreased productivity and neglected wages. While there are numerous causes of lower back pain, IVD degeneration and its sequelae, such as disc herniation, accounts for 40% of cases [2]. The nucleus pulposus (NP), annulus fibrosis (AF), and cartilaginous endplates (CEPs) all contribute to the structural stability of the IVD. Among these components, the NP plays a critical role in homeostasis maintenance by producing a complex extracellular matrix (ECM) (type II collagen and proteoglycans). This ECM composition is required for the IVD’s physiological viscoelastic characteristics. Notably, IVD degeneration is generally thought to begin with changes in the NP with a host of well-studied changes in ECM elements [3]. While also caused by other numerous different pathoanatomic findings, degeneration of the intervertebral disc’s annulus fibrosus, followed by extrusion of the nucleus pulposus material and subsequent compression of nerve fibers, is a leading cause of this lower back pain [4]. Degeneration of IVD begins with an increase in proteolysis and inflammatory cytokines, while aging and alterations are associated with the breakdown of extracellular matrix, particularly aggrecan and collagen. Simultaneously, age-related degeneration of CEPs, such as sclerosis or increased thickness, leads to decreased permeability and limited nutrition transfer, resulting in IVD cells’ failure to synthesize and maintain the appropriate ECM. As a result, NP-transmitted stress causes excess stress concentrations and increased shear stress in the AF, increasing the probability of structural damage, often resulting in bulging and fissure formation. The fissures further create a suitable microenvironment for the deeper ingrowth of nociceptive nerve fibers and vascular invasion from the AF’s outer layers, resulting in discogenic low back pain [5]. Also, as a distinguishing element, the inflammatory response is also an essential signal in the degenerative cascade, resulting from an imbalance of anabolic and catabolic factors. For example, the interleukin family (e.g., IL-1b, IL-1, IL-4, IL-6, IL-12, IL-17, MMPs), interferon gamma (IFN-g), and tumor necrosis factor-a (TNF-a) produced by inflammatory cells trigger overexpression of matrix metalloproteinases (MMPs), including MMP-1, MMP-2, MMP-3, and MMP-7. The production of these matrix metalloproteinases, in turn, accelerate collagen and proteoglycan catabolism. Furthermore, IL-1b increases the expression of vascular endothelial growth factor (VEGF), nerve growth factor (NGF), and brain-derived neurotrophic factor (BDNF), worsening IVD degeneration and the associated symptoms [6]. Minimally invasive regenerative procedures, complete biologic disc replacement, and cartilaginous endplate decalcification are all urgently needed regenerative techniques [7]. Currently, both surgical and non-surgical therapies are the mainstays of treatment and include physical therapy, anti-inflammatories, corticosteroids, and surgery to arthrodesis or replace the symptomatic IVD. However, while effective, these therapies have their limitations as they are largely ablative, with an emphasis on reducing the patient’s pain and dysfunction, and are neither able to reverse the IVD degenerative process nor restore the mechanical and physiologic function of the IVD [4,8]. 

Many recent strategies have focused on composite hydrogels, which provide greater control over the regenerative strategy by combining different degradable or non-degradable polymers that (a) interact with host tissues, (b) replace functional tissue by mimicking morphological characteristics of natural systems, and (c) stimulate the healing process. In this context, hydrophilic materials and hydrogels, in combination with other material phases tightly structured in composite formulations, can successfully meet the basic criteria of natural soft and hard tissue transport and mechanical qualities [9]. Consequently, novel research has revealed hydrogels as a promising therapeutic for intervertebral disc degeneration that may overcome the limitations of current treatment options.

Hydrogels are a group of three-dimensional polymeric materials with an extensive hydrophilic structure, which allows for them to hold an abundant amount of water [10]. Many hydrogels are readily compatible with biological tissues and do not elicit major immune responses. Additionally, because of their high water content, hydrogels can also offer a suitable aqueous environment that promotes the proliferation and activity of IVD cells [4,5,6]. These biomaterials also have a variety of tunable biochemical and biomechanical properties, such as shear-thinning behavior, biodegradability, ECM biomimicry, and physiochemical properties that make them an attractive, appropriate, and functional tool to address intervertebral disc degeneration [11]. Through various studies on hydrogels as therapeutics, it has been found that these soft biomaterials may be able to serve as injectable carriers of cells and/or biomolecules to be delivered to injury sites and restore tissue function [10,11,12]. Due to the immense promise hydrogels possess, a substantial amount of research has been carried out in the last two decades to evaluate this class of biomaterials. For instance, an early study by Reitmaier et al. evaluated two hydrogels functionalized with anti-angiogenic peptides and seeded with bone marrow-derived mononuclear cells (BMC) in an ovine nucleotomy model [13]. While this study did not find either of the hydrogels capable of regenerating the biofunctionality of the native intervertebral disc (likely due to hydrogel extrusion), the importance of the development of annulus sealants was illuminated [13]. As such, this finding determined an important component in generating a successful synthetic to combat IVD degeneration. The GelMA hydrogel composite technology [14] demonstrates a novel approach that builds on previous studies, such as that done by Reitmaier et al. The GelMA hydrogel has the potential to improve current clinical treatment approaches, acting as filling agents and scaffold materials for tissue defects, enabling regenerating by supporting IVD cell functional metabolism and acting as a biomechanical component in IVDD treatment.

Accordingly, in this review we will discuss the comprehensive development of hydrogel-based strategies for intervertebral disc regeneration from initial work in the laboratory to clinical perspectives. We will also seek to explore the latest advancements and build upon the previous literature, ultimately illuminating how novel hydrogel strategies have significantly advanced. This review was done via a comprehensive search of publicly available databases for research articles in areas that have shown significant promise including, but not limited to, biological components in hydrogel formulations, hydrogel fabrication and characterization, in vitro studies, in vivo preclinical models, clinical trials, and translational research. Additionally, this review will address the less explored areas of challenges and future directions, as well as regulatory and ethical considerations.

## 2. Anatomy and Pathophysiology of the Intervertebral Disc

**Figure 1:**
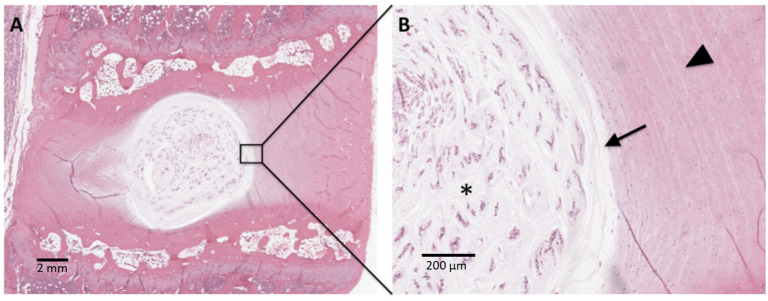
Low (**A**) and high (**B**) power H&E-stained histological section of uninjured IVD from a rabbit pre-clinical model. The NP (*) is populated by clusters of cells within a gelatinous matrix. A clear border (arrow) between the NP and AF is evident. The AF demonstrates organized fibrocartilage lamellae (arrowhead).

The intervertebral disc is an essential component in the function and maintenance of the spinal column. These fibrocartilaginous, articulating structures are located between vertebral bodies and anterior to the intervertebral neuroforamen [15,16]. Structurally, the intervertebral disc can be divided into the tough and highly organized outer annulus fibrosus, and the soft and gelatinous inner nucleus pulposus, shown in Figure 1.

The nucleus pulposus is composed predominantly of charged proteoglycan molecules mixed with Type II collagen, which attracts and holds water within the structure, giving the nucleus its soft, gelatinous consistency [16]. The high water content of the nucleus pulposus allows for it to distribute hydraulic pressure throughout the IVD, thereby dispersing the compressive forces that the spinal column endures during normal locomotion [16]. The annulus fibrosus, on the other hand, has a structure that is highly organized, consisting of 15 to 25 stacked sheets of predominantly collagen, with interspersed proteoglycans, glycoproteins, elastic fibers, and the connective tissue cells that secrete extracellular matrix material [15,16]. The annulus is further divided into an inner and an outer portion. While both are primarily collagen, the outer annulus contains mostly type I collagen, while the inner has a higher type II collagen content, as well as a greater number of proteoglycans [16]. This tough structure allows for the annulus to encircle and confine the gelatinous nucleus pulposus. In addition, the rigid structure allows for increased resistance to the compressive forces exerted upon it by the nucleus pulposus, while also resisting torsion, flexion, and extension movements of the spine [16].

While these structural components allow for the intervertebral disc to withstand a variety of forces, degeneration can (and often does) occur. The complex interplay between various cellular-level processes often begins with an imbalance between catabolic and anabolic processes, followed by extracellular matrix degradation, neo-innervation, and neovascularization [17]. As a result, this degenerative process results in loss of the nucleus pulposus, loss in disc height, and subsequent disc bulging [17]. This weakened structure can lead to a variety of symptomatic pathologic conditions including disc herniations, spinal stenosis, spinal instability, and deformity [4,17].

## 3. Biomechanics and Material Requirements for Intervertebral Disc Regeneration

As described, biomechanically the intervertebral disc functions in maintenance of normal musculoskeletal physiology. An effective biomaterial that could replace the intervertebral disc following degeneration would require sufficient mechanical properties to be able to promote longevity, reduce re-herniation risk, have tensile strength, have a fair amount of compressive and shear force resistance, axial and torsional biomechanical properties, in vivo compatibility, and an ability to restore intervertebral disc height [18]. 

Various studies have found that hydrogels possess several of the aforementioned key characteristics. For example, Li et. al. found a three-dimensional cell encapsulating synthetic hydrogel that mimicked the native intervertebral disc structure and function, as well as provided a suitable environment for cell survival [19]. Similarly, Cheng et al. determined that a hydrogel scaffold loaded with microspheres had good mechanical properties and a low immunogenicity that allowed for a restoration of the biomechanical properties of the intervertebral disc, while minimizing the incidence of re-herniation events [20]. These studies’ outcomes, in conjunction with outcomes from a variety of other studies, make it evident that hydrogels hold a great degree of potential as viable intervertebral disc replacement options following disc degeneration events. 

## 4. Hydrogels: An Overview

As discussed, conservative and surgical treatment options for intervertebral disc degeneration include anti-inflammatory analgesics, physical therapy, epidural injections, surgical decompression, disc replacement, and vertebral fusion [21]. While effective, these conventional therapies do have their limitations and, as a result, other more comprehensive treatment paradigms have been investigated. One such area that has yielded many satisfactory results in recent years is tissue engineering [22]. A significant amount of research is in progress for the use of hydrogel implants in the treatment of IVD degeneration, as, in addition to providing an environment of cellular function and proliferation, they can readily be constructed to reproduce the mechanical characteristics of native nucleus pulposus tissue. 

Hydrogels are three-dimensional hydrophilic polymers known for their high biocompatibility and high water content [23,24]. Hydrogels with 3D cross-linked network architectures, customizable physicochemical properties, and similar extracellular matrix topologies for cell adhesion and proliferation have received a lot of attention. Hydrogel designs consider implant performance in terms of structural integrity, biocompatibility, biodegradability, safety, cellular solute transport, mechanical strength, and viscosity. In this regard, because hydrogels vary in material type, molecular weight, crosslinking degree, chemical surface, solid contents, and functionalization, they can be formulated to mimic the mechanical properties of native tissues. Furthermore, most of these hydrogels can be implanted in vivo with minimally invasive techniques, obviating the need for invasive procedure for implantation. Hydrogels are being studied for a variety of applications in the treatment of IVD degeneration as scaffolds for carrying cells, slow delivery release systems for bioactive molecules (drugs, small molecules, and growth factors), templates for extracellular matrix deposition, disc rehydration, and any combination of these. Ideally, an injectable, in situ curable hydrogel would aid in the restoration of disc structure and function by establishing a protective microenvironment conducive to cell proliferation, extracellular matrix deposition, and the gradual release of bioactive chemicals within the diseased disc’s non-vascular milieu. The existing mechanical IVD prostheses [23,24,25] can only be used to replace the nucleus pulposus and are unable to promote the regeneration and repair of nucleus pulposus tissue. Additionally, they restrict their application due to the risk of complications from prosthesis displacement and the implantation process [25]. As a result, the research focus has shifted to the creation of biologically active hydrogels with simple compositions and synthesis for tissue regeneration. 

## 5. Biological Components in Hydrogel Formulations

Hydrogels are both synthetic and natural biomaterials that have demonstrated, through extensive research, the ability to serve as a potentially viable replacement option for the intervertebral disc following degeneration [10,11]. As they are meant to serve the various roles of the intervertebral discs, these hydrogels must be synthesized in a specific manner and with specific biological components based upon the type of hydrogel desired. 

Yan et al. and Ying et al. have characterized synthetic hydrogels as three-dimensional network microstructures that are formed by the combination of hydrophilic molecules that initiate a sequence of complete hydration and swelling [12,26]. These biomaterials vary in their composition and include substances (or derivatives of these substances) such as polyacrylic acid, polyacrylic acid salts, polyacrylamide derivatives, and polyvinyl oxide, to name a few [12,26]. Synthetic hydrogels have the advantage of being able to overcome the often-insufficient mechanical properties of natural hydrogels [12,26]. Despite this major advantage, synthetic hydrogels also have several limitations that include the addition of potentially toxic components in the process of preparation, generally slower degradation, and often insufficient native biologic activity [12,26]. Consequently, synthetic hydrogels are rarely used alone in tissue engineering, and are instead utilized in conjunction with natural hydrogels to formulate a robust and optimal replacement biomaterial [12,26]. Studies on these synthetic hydrogels and their outcomes are summarized in Table 1. 

Alternatively, natural hydrogels are usually derived from animal and plant extracts. According to Yan et al. and Ying et al., commonly used natural hydrogels include gelatin, collagen, fibrin, hyaluronate, alginate, agarose, and chitosan [12,26]. Due to their derivation from naturally occurring extracts, these hydrogels are nontoxic, have a high degree of biologic safety, and possess a high level of biocompatibility, allowing for a more natural integration into patients that receive these as a replacement [12,26]. Accordingly, owing to their significant biocompatibility, natural hydrogels are widely used in tissue regeneration and repair [12,26]. Studies on natural hydrogels for IVD regeneration are summarized in Table 2.

Moreover, a novel hydrogel type that has recently shown to have substantial potential is the composite hydrogel [28]. Composite hydrogels are composed of a combination of both synthetic and natural polymers [28]. Bioengineering these composite hydrogels from natural and synthetic hydrogels is still a relatively new concept and much research has been moving in this direction, with the goal of formulating a polymer that is able to provide the best possible intervertebral disc replacement [28]. Current formulations are shown in Table 3.

It is evident that hydrogel synthesis involves the integration of many bioactive components in both natural and synthetic hydrogel formulations. These bioactive materials allow for these hydrogels to exhibit a range of properties that allow for regeneration of properties that make the intervertebral disc both unique and essential [12,19,42,43,44,45,46,47,48,49,50,51]. Correspondingly, an understanding of the roles of these bioactive materials is crucial and allows not only for an understanding of the potential these hydrogels can have, but also help to progress this area of biosynthetics, thereby generating biomaterials that are more like native intervertebral disc material. 

## 6. Hydrogels in Intervertebral Disc Tissue Regeneration 

Hydrogels with 3D cross-linked network architectures, customizable physicochemical properties, and comparable extracellular matrix structures for cell adhesion and proliferation have received a lot of attention in intervertebral disc regeneration therapy. Naturally-derived hydrogels, such as Chitosan [34], Alginate [36], Hyaluronic acid [52], Collagen [53], and Gellan gum [54], are particularly appealing due to their inherent biocompatibility, biodegradability, and safety. These hydrogels are derived from renewable resources such as animal, plant, algae, and microorganisms found throughout the world. Synthetic hydrogels, which primarily contain Polyethylene glycol (PEG) [55], Polyurethane (PU) [56], Polyvinyl alcohol [57] and Poly(lactic-*co*-glycolic) acid [58], have tuneable qualities that allow for the simple creation of functional products. To ensure the biosafety of synthetic hydrogels, it is essential to eliminate impurities, unreacted chemicals, surplus monomers, catalysts, and other by-products [3]. In this regard, because hydrogels differ in material type, molecular weight, crosslinking degree, chemical surface, solid contents, and functionalization, they can be used to replicate the mechanical qualities of natural tissues. Furthermore, their crosslinked structures may exhibit tissue-like viscoelasticity, diffusive transport, and interstitial flow properties. Table 4 summarizes the recent advancements and approaches of hydrogel-based biomaterials for the intervertebral disc.

### 6.1. In Vitro Outcomes

With the immense potential of hydrogels, extensive testing has been conducted in vitro with a variety of different formulations. Research has largely been performed with hydrogels that fall into two categories: those that are pre-formed prior to implantation, and those that are cross-linked in situ (injectable). In conducting studies testing pre-formed hydrogels, cells first had to be seeded onto previously cross-linked discs. In going through this process, cellular penetration can be difficult to obtain, and distribution is not always consistent. Following seeding, surgical exposure of the IVD is necessary and an incision in the AF would be required to implant the scaffold into the spine. This process increases the invasiveness of the treatment strategy and carries the risk of construct ejection into the spinal canal or other anatomic compartments. On the other hand, injectable hydrogels involve a significantly less invasive process, where only a needle-sized defect through the AF is necessary, the injected material can conform to the shape of the defect, and the cells, loaded medications, chemicals, and proteins are able to be distributed evenly throughout the tissue. These injectable hydrogels also have the advantage of being immobile and having a lesser risk of extravasation once it is cross-linked in situ. 

Because of the intervertebral disc’s lack of vascularity and low cell density, extracellular matrix-based biomaterials in hydrogel form become the most advantageous platform for creating 3D scaffolds while keeping their biological features. For instance, A collagen II hydrogel has increased cell viability without affecting the NP phenotype [71], and on the other hand, microencapsulation of NP cells in the 3D microspheres system of collagen I demonstrated a round morphology of NP cells that maintained NP phenotypic markers of type II collagen and cytokeratin-19 [72]. Additionally, collagen type II cross-linked with genipin has been used to promote the differentiation of adipose-derived stem cells (ASDCs) into NP-like cells through Shh pathway [73]. In addition to this, genipin has also been used to stabilize a collagen type II and chondroitin sulphate gel for NP-like expression in ASDCs and partial restoration of NP [74]. Similarly for the AF, scaffolds collagen type I [75,76] with the addition of cells and growth factors [41] are commonly preferred. 

Recent research was conducted on reversing the AF degradation in sheep with an MSC-laden hydrogel [77], and AF-derived stem cells have been trialed in a collagen type I-containing decellularized ECM (dECM) [78]. Furthermore, tissue-specific dECM is considered as a promising alternative, as these constructs enable a more accurate reflection of native tissue environment [79,80], delivering collagens, proteoglycans, and other important matrix components [81]. In the case of the IVD, dECM biomaterials have been used to provide scaffold materials, and MSCs co-cultured with degenerate NP cells [82] and used as scaffold coatings within hydrogels and for bioinks in 3D bioprinting [83,84,85,86,87,88]. 

In the near future, the research on this scope will rely on combined collagen type I and collagen type II blended hydrogel [89] with chondroitin sulphate [90] for MSC differentiation and use of combinations of matrix molecules simultaneously in NP, AF, and CEP. Proteoglycan-like systems vitally mimic heparan sulphate [91] and chondroitin sulphate chains of aggrecan to confer a hydrating function [92,93], aid in differentiation [94] and deliver growth factors to MSCs [95], encourage collagen and GAG production in MSCs for NP regeneration [96]. 

There are few attempts in the use of electrospun systems incorporating biochemical and physical matrix cues within an IVD context [97]. Electrospun fibers combined with dECM are promising for cartilage tissue engineering, but further study for application to IVD is needed [98]. The fabrication of electrospun IVD systems depends on manual rolling of the AF and injection of an NP-like hydrogel which can introduce discontinuities between IVD regions [99,100,101] based on the variable stiffness these electrospun materials. Conventionally, many electrospun constructs have been implanted and studied in small and large animal models [39,76,102] for the synthesis of total disc replacement devices and other tissue engineered approaches. 

Another scalable approach to model the NP and AF is 3D bioprinting, which includes techniques such as vat polymerization, material jetting, and extrusion-based bioprinting, allowing precise delivery of cells and other components to materials with complex architecture [23,103,104,105,106,107]. The challenge in achieving shape fidelity while printing hydrogels is addressed by adopting strategies like Freeform Reversible Embedding of Suspended Hydrogels (FRESH) and Suspended Layer Additive Manufacturing (SLAM) to restrict fluid flow and improve printability [108,109,110,111]. To print stiffer AF region hydrogels, materials such as gelatin and gellan gum based have been co-printed with PCL [112,113]. Bioprinting of hydrogel scaffolds that mimic the AF’s lamellar structure and NP interface [114] have been attempted, with the prospect of employing patient-specific MRI data to create more accurate IVD models [115,116]. Overall, these studies demonstrate new opportunities for ECM engineering that have the potential to alter the degenerate IVD microenvironment. Table 5 summarizes the recent approaches and their in vitro findings of hydrogel-based biomaterials for the Intervertebral Disc Degeneration. 

### 6.2. In Vivo Pre-Clinical Models

In exploring and researching hydrogels as intervertebral disc material replacements, many of the studies have predominately been in vitro; however, with the promising results seen in in vitro trials, several studies have progressed to in vivo animal trials. Studies that investigate hydrogels for intervertebral disc replacement are summarized in this section.

One such study investigated the reparative effect of platelet-rich plasma and ferulic acid hydrogel compounds on degenerated IVDs in rats. Chai et al. found that this hydrogel formulation was injected, and outcomes observed included histomorphology, apoptosis, and protein synthesis of the intervertebral disc [125]. From this study, it was found that the factor release concentration of all groups peaked at 12 h [125]. In addition, it was found that this hydrogel formulation was cytocompatible and ultimately, that this PRP/FA-rich hydrogel compound plays an active role in promoting extracellular matrix synthesis, strengthening, and repairing degenerated intervertebral discs in rats [125].

Furthermore, Inoue et al. found that injection of a hyaluronic acid hydrogel promoted intervertebral disc repair in a rabbit model of IVD degeneration [126]. This study found in the experimental group with the hyaluronic acid injection, disc height was increased at weeks four and eight [126]. The experimental group that received the hyaluronic acid hydrogel was also seen to have a significantly downregulated inflammatory response (specifically downregulation of IL-6), as well as a slightly reduced IL-1β and TNF-α response [126]. Consequently, it was evident that an injectable hyaluronic acid hydrogel had protective effects in slowing down disc height loss, promoting tissue hydration and thereby enabling intervertebral disc repair, and attenuating inflammation and innervation to prevent further disc degeneration [126].

Watanabe et al. also found that intra-discal injection of a hyaluronic acid hydrogel derivative had restorative effects in a rabbit experimental model [127]. Watanabe et al. punctured two rabbit intervertebral discs to mimic disc degeneration, followed by injection with either the hyaluronic acid hydrogel derivative or normal saline. Several outcome variables were evaluated including disc hydration, height, appearance, tissue organization, and safety [127]. From the study, it was determined the hyaluronic acid hydrogel injection restored disc height to over 75% of the pre-punctured disc, improved water retention, and restored normal disc appearance in nearly 84% of experimental subjects [127]. In addition, tissue organization and cellularity in the hyaluronic acid derivative hydrogel group resulted in significantly lower intervertebral disc degeneration scores than saline (*p* < 0.01) [127].

While these in vivo studies have largely focused on hydrogels to restore the anatomy and physiology of the intervertebral disc, many in vivo studies have investigated hydrogels as delivery systems meant to modulate the immune system and to promote natural IVD regeneration. This was seen with Li et al.’s recent successful development of a fucoidan-DexMA composite hydrogel scaffold to regulate ECM metabolic homeostasis, inhibit inflammation, and restore tissue function [128]. Through numerous in vivo and ex vivo experiments, the team was able to determine that this hydrogel scaffold increased the proliferation of nucleus pulpous cells (NPCs) whilst enhancing the synthesis of ECM on NPCs via the CAV1-YAP signaling pathway [128]. The scaffold was also seen as capable of promoting the M2 polarization of infiltrating macrophages and effectively alleviating the inflammatory microenvironment [128]. Correspondingly, intervertebral disc degeneration was significantly decreased, and tissue regeneration was effectively promoted [128]. Similarly, Yu et al. developed a biocompatible polyurethane scaffold loaded with fucoidan (F-PECUU) for ameliorating the degenerated IVD microenvironment to promote regeneration [129]. The F-PECUU hydrogel scaffold also alleviated the inflammatory and oxidative stress caused specifically by lipopolysaccharides and prevented extracellular matrix (ECM) degradation in AF cells, while promoting ECM deposition to maintain the height, water content, and mechanical properties of IVDs in in vivo testing [129].

An additional study that found success with utilization of hydrogels as a means of immunomodulation was a recent study by Yuan et al. that developed and injected biodegradable microspheres and hydrogels containing Etanercept (ETN), a TNF inhibitor currently in clinical use for inflammatory conditions, and Growth Differentiation Factor 5 (GDF5) [130]. Through injection of these biospheres in the rabbit disc puncture pre-clinical model, team was able to determine that these biospheres were biocompatible and were able to deliver sustained and therapeutic dosages of ETN and GDF5 [130]. This sustained combination of therapeutics resulted in a long-term anti-inflammatory and regenerative effect in the IVD [130].

While these six in vivo studies highlight advances that have been made within recent years, there exist still numerous other studies, which are depicted in Table 6 below.

The safety and efficacy of these biomaterials are encouraging, as evidenced by the promising results seen in restoration of disc height, hydration of intervertebral disc material, and reduction the inflammatory response that led to initial degeneration of the intervertebral disc [108,109,110,111,112,113,114,115]. Correspondingly, there should be a movement towards human clinical trials, and eventual implementation as a mainstay of treatment in orthopedic spine treatment and care.

### 6.3. Clinical Trials and Translational Research

There has been a surge of interest in the paradigm shift away from surgical intervention and toward biological therapies that promise to restore disc morphology and function in recent years. The ability of bioengineered therapeutics to stimulate multi-tissue regeneration processes even within mechanically demanding environments such as the spinal column underpins these emerging concepts. Many scientific studies conducted over the last two decades have discovered crucial signaling pathways, as well as potential chemicals and pluripotent cells that could be therapeutically useful. However, it is still unclear how many of the published preclinical trials can be translated to clinical use. Indeed, despite the abundance of research focusing on biological therapies to restore disc anatomy and mechanical qualities, just a few bioengineered treatments are now undergoing clinical trials (according to publicly available data). There are clinical trials reported to involve growth factors or recombinant human growth/differentiation factors, peptide and corticosteroid injections, as well as cell implantation techniques to promote disc tissue repair. However, published evidence and success rates in human trials is very limited.

The fundamental problem in clinical fields is that decision making is mostly influenced by the patient’s report of pain and functional impairment, as opposed to identification and reversal of the pathoanatomic process of IVD degeneration. In many patients, there is poor correlation between clinical symptoms and pathoanatomic degeneration. However, the efficacy of current initiatives to advance regenerative technology have been judged by the achievement of biological goals, rather than clinical improvement. Apart from more effective in vivo non-destructive imaging technology, biomarker testing may be required to understand the biochemical cues underlying the morphological and functional condition of the degenerative disc. The immediate future, therefore, lies in the ability of the scientific community to achieve the synthesis of personalized treatment strategies that should be adjusted to regenerate the damaged disc, with the goal of improving future management of clinical symptoms with a better outcome than the present therapeutic arsenal. Furthermore, with an accurate diagnosis of patient-specific pain generators, individualized therapy will be possible. It is time to transfer the underlying science of biologic-mechanistic intervertebral disc into effective therapies capable of reducing discogenic lower back pain in patients.

## 7. Challenges and Future Directions

While the capacity of hydrogels to serve as a better treatment alternative in patients with intervertebral disc degeneration cannot be overlooked, as with any novel therapeutic, there remain several current challenges in moving this therapeutic strategy forward.

One study that highlights some of these challenges is the investigation of a tissue-engineered injectable gelatin–methacryloyl (GelMA) hydrogel-based adjunctive therapy [132]. From their examination, Li et al. found that while these hydrogels did induce disc tissue repair, the therapeutic effect of this biomaterial was heavily concentration dependent [132]. In addition, it was found that the degradation rate of in vitro pure GelMA hydrogel is relatively fast [132]. As such, these two challenges make the GelMA hydrogel unable to meet the requirements of cell growth and proliferation [132]. Furthermore, because of the way in which the GelMA hydrogel (and various other hydrogels) are fabricated, they may be recognized as a foreign substance within the human body and may lead to immunologic rejection of the therapeutic material [132].

Likewise, through review of several studies investigating the repair of degenerated intervertebral discs, Ying et al. found that depending on the type of hydrogel utilized, a unique set of difficulties arise [26]. The team found that synthetic hydrogel repair strategies are poorly biocompatible, despite having advantages in terms of mechanical properties [26]. Alternatively, natural hydrogel repair strategies lack ideal mechanical properties; however, they do more reliably maintain cell activity, promoting cell proliferation and differentiation, as well as extracellular matrix synthesis, and maintaining the water content, and thus the mechanical health, of nucleus pulposus [26]. As a result, while each of the hydrogels do confer a unique advantage, no single type has been seen to exhibit all the properties of a native intervertebral disc, thereby stalling progression of this therapeutic to future clinical trials and implementation.

While we highlight a few of the main challenges, several others do exist. This begs the question, at this current stage, of how we may overcome these challenges and continue to progress forward in development, and eventually implement this therapeutic as a mainstay of treatment? While further research and testing is necessary to demonstrate the efficacy and power of these hydrogels, numerous studies propose further exploration of composite scaffolds built from both synthetic and natural hydrogels [18,19]. In this way, the advantages of both individual hydrogel types can be utilized as an effective replacement option for the intervertebral disc [18,19]. Additionally, as discussed above, Ying et al. found that one of the biggest obstacles that may be encountered with hydrogel therapy is risk of graft rejection [26]. In overcoming this challenge, more studies should investigate not only the safety of the biomaterial when injected, but also the long-term consequences and effects of the hydrogel. Furthermore, the metabolism and metabolic properties of hydrogels is another area that should be explored [26,127,132]. In doing so, the half-life and concentration of the biomaterial can be optimized so that it remains in patients with intervertebral disc degeneration for an appropriate length of time, thereby conferring a stronger and longer-lasting therapeutic effect.

## 8. Regulatory and Ethical Considerations

Despite the tremendous biologic potential of hydrogels for IVD therapy, as with nearly all novel therapeutics, there exist many regulatory and ethical considerations concerning the approach. Throughout this review, it has been demonstrated that all forms of hydrogels require an extensive bioengineering process, potentially involving multiple biologically active compounds [26]. While tested substantially in vitro and in pre-clinical animal models, there has been limited human testing performed. Shepherding these complex composites through the regulatory process can pose a significant challenge.

In addition to immediate toxic or immunogenic effects, another important regulatory and ethical element is the concept of the long-term effects of hydrogel injections. Throughout lab testing and in animal models, hydrogels have not been shown to have cell toxicity and have been well tolerated [19,27,125,126,127]. However, these hydrogels have been tolerated in the short term, with many of these studies collecting follow-up data spanning only weeks or months. This limits our understanding, not only of the long-term effects of hydrogels, but also of how these elements integrate with human tissue over time, as well as the viability and efficacy of these injections in conferring a therapeutic effect over time.

Finally, perhaps one of the most important ethical and regulatory considerations to be aware of over the near future is the cost and availability of this novel therapeutic. According to Mandal et al. the cost of development of a hydrogel through clinical translation ranges anywhere from $50–800 million [133]. This enormous cost into research and development eventually will translate to an immense cost to patients and the overall healthcare system, an issue which is compounded by the large number of patients with symptomatic IVD degeneration.

The issues of cost, long-term effects, and safety in humans are not insignificant issues and illustrate a few of the regulatory and ethical issues that must be considered. Accordingly, research in this area will need to take into consideration these, as well as other ethical and regulatory aspects, in the formulation, development, and implementation of hydrogels as a mainstay of therapy.

## 9. Conclusions

Currently, approved treatment options for the extremely common conditions resulting from IVD degeneration are limited, the most popular options being conservative treatment, corticosteroid injections, physical therapy, and surgical intervention [8,27]. While often effective, these therapies have their limitations as they are based upon the patient’s symptoms, with an emphasis on correcting the patient’s pain. These therapies therefore lack the ability to reverse the underlying degenerative process or restore the biomechanical function of this unique and important tissue [4,8]. As the incidence of IVD degeneration has increased, the pathophysiology of this disease has been increasingly studied, and various treatments have been developed. Hydrogels have been demonstrated to potentially be one of the most effective and viable therapeutic options, with the capacity to mimic native tissue structure and function [10,11,18]. Numerous studies have found that various hydrogel formulations have biomechanical properties and can be incorporated with bioactive substances to allow for functional therapeutic action while sustaining the forces that the human spine encounters during normal function [19,20]. This understanding of hydrogels, as well as the intervertebral disc anatomy and physiology, has translated to the success of various in vitro and in vivo studies, providing a framework for how scientific research in this area can continue to move forward [19,23,24,26,110,112,114,125,126,127].

However, even with the growing wealth of knowledge in this area, further challenges, questions, and regulatory and ethical considerations have come to light. There is a great deal of uncertainty surrounding the clinical translation of these hydrogels, requiring substantial further research to eventually reach human subject testing and clinical trials. While these challenges exist, it is evident from this literature review, that hydrogels have immense potential in truly slowing and/or reversing IVD degeneration. While significant strides have been made in advancing this therapy as a viable option, it is necessary to continue to move forward and further understand, characterize, test, and eventually implement hydrogel therapy into strategies to address an important and costly clinical challenge.

## Figures and Tables

**Table 1:** Summary of current synthetic hydrogels advantages and disadvantages, and material degradability in in vitro studies.

Material	Component	Advantages	Drawbacks	Material Degradability	References
Biodegradable electrospun PCL membrane	Polypractalone (PCL)	Slow degradation; good for long-term repair	None reported	Slow degradibility over degradation period. Scaffolds tend to shrink during period independent of temperature.	[26,27,28]
PVA combined with Silicone Rubber	PVA; Silicone Rubber	Biomechanical properties like human NP	None reported	None reported	[26,29]
Copolymers of TMC and HDI	Polyethyleneglycol; TMC; HDI	Excellent adhesion and cytocompatibility	Poor mechanics	Copolymers had low degradation rates over the course of 3 weeks in vitro.	[26,30]
U2000-2-prepolymer	Polycarbonatediol; Hexamethylene diisocyanate monomers	Excellent adhesion; covalent binding with gelatin without the catalyst	Sterility biomechanics and biocompatibility unknown	In subcutaneous locations, tissue residues were found after 12 months and in intramuscular locations, tissue degradation was complete after about 6 months. Good biocompatibility throughout different degradation stages. After full degradation, complete remodeling to physiological tissue was observed.	[26,31]
PVA/PVP hydrogel	PVA; PVP	Excellent biomechanics	None reported	Total loss of cross-linked hydrogels was found to be significantly lower than for native hydrogels.	[26,32]
PEG combined with DNCM	PEG; DNCM	Excellent biocompatibility	Requires further biomechanical adjustment	Polymer was designed to mimic slow degradation under physiological conditions to promote a slow replacement of hydrogel with newly deposited matrix within the IVD, as NP matrix turnover takes months to years.	[26,33]

**Table 2:** Summary of current natural hydrogels advantages and disadvantages, the study design utilized, and material degradability.

Material	Component	Advantages	Drawbacks	Material Degradibility	References
Novel temperature-sensitive chitosan hydrogel	Chitosan; sodium bicarbonate	Mechanical properties resembling human IVD; NP cell activity maintained; promotion of GAG production	None reported	All proposed hydrogels exhibited enhanced mechanical and degradation properties; one formulation (containing 2% chitosan, 7.5 mM of SHC, and 0.1 M of BGP) showed properties most similar to native human NP tissue.	[26,34]
Decellularized ultra-purified alginate gel	Alginate	Facilitated ECM generation; proliferation of endogenous NP progenitor cells	Lack of safety tests for immunogenicity; not as suitable for older patients	None reported	[26,35,36]
ChABC-treated collagen gels	Collagen	Enhance adhesion	Safety needs to be clinically tested	Stable and slow degradation at low temperatures; however, hydrogel stability is questionable at physiological temperatures	[26,37]
Nanostructured gelatin colloidal hydrogels [38]	Gelatin [38]	Shear-thinning; self-healing; adjustable mechanical properties; preservation of cell activity and supported NP- like differentiation of BMSCs [38]	None reported	Biodegradation of gelatin colloidal gels reported with a degradation rate of over 70% within 4 weeks; however, ideal degradation rate of the colloidal gels with regard to inducing IVD regeneration unknown.	[26,38]
High Density Collagen Gels	Collagen	Water content of NP maintained [39]	Require a longer follow up period; lack of biomechanical testing	Collagen hydrogels have been seen to have poor degradation properties; several strategies have been hypothesized to enhance their degradation properties, but they are in infancy.	[26,39,40]
Porcine Fibrin Gel	Fibrin	Water content of NP maintained	Lack of molecular level studies	Biodegradable; results showed that collagen I hydrogel maintained AF cell survival after 7 d of 3D culture in vitro but did not further enhance cell proliferation After 14 d of organ culture under daily dynamic loading, PU-Col constructs could still completely fill the AF defect and no herniation of NP tissue was found. After 14 d of in situ culture with daily dynamic load, the implanted AF cells remained in the PU-Col constructs and started matrix production.	[26,41]

**Table 3:** Summary of current composite hydrogels advantages and disadvantages and the study design utilized.

Material	Component	Advantages	Drawbacks	References
FibGen	Genipin and Fibrin	Better axial compression performance; improved tensile stiffness and axial neutral zone length of IVDs; restoration of water content of IVD; successful delivery of cellular and biological material	Axial normal levels not able to be restored	[26,42,43]
Injectable Photocurable Hydrogel	PEGDA; (DAFM)	Maintenance of porous structure; ECM deposition	Lack of specific markers for AF	[26,44]
HA injection + Crosslinked photocrosslinked collagen patch	HA photocrosslinked collagen patch	Biomechanics and hydration of IVD recovery	Functional analysis not focused on kinematics of entire spine	[26,45]
Nanocomposite hydrogel	Methacrylated gellan-gum; Cellulose nanocrystals	Mechanical properties enhanced; activity of IVD is close to natural IVD	None reported	[26,46]
g-DAF-G	Genipin; Decellularized AF	Differentiate human BMSCs to AF cells; excellent bioactivity and cellular extensibility; water content of NP restored	Method of induced regeneration is inefficient	[26,47]
PCL-Supported Electrocompacted Aligned Collagen Type-I Patch	PCL; Collagen	Recovery of IVD biomechanics; produce type I collagen and GAG; promotion of proliferation of AF cells	None reported	[26,48]
Cellulose Nanofiber Reinforced Chitosan Hydrogel	Cellulose Nanofibers; Chitosan	Enhance the mechanical properties; activity of this IVD was similar to natural IVD	Non-cellular repair	[26,49]
Injectable Interpenetrating Network Hydrogels	Fibronectin conjugated fibrin; poly-(ethylene glycol) diacrylate; doubly-modified GAG	Non-cytotoxic; easily apply to AF defects in a short time	Immune responses, changes in pain, in vivo degradation, endogenous cellular repair was not assessed	[26,50]

**Table 4:** Recent advancements and approaches of hydrogel-based biomaterials for the intervertebral disc regeneration.

Materials	Encapsulated Agent	Functions	Major Findings	Reference
PEG-LM (peptide-functionalized poly(ethylene glycol) (PEG)-based hydrogel)	Primary NP cells	Promotes ECM secretion; promotes cell proliferation	The PEG-LM promotes cell viability, increased biosynthetic activity, and NP-specific protein expression in both in vitro and in vivo models. It can contribute to the restoration of disc height and endplate organization. The cell-laden hydrogel delivery system shows improved disc phenotype, including organized endplates, observable lamellae, and increased cellularity within the central region of the disc. It demonstrates effective delivery and retention of NP cells into the intradiscal space.	[59]
ASP-liposomes @ gelatin methacryloyl (GelMA) hydrogels	Aspirin	Relieves local inflammation	ASP-Lip@GelMA hydrogel treatment effectively decreased inflammatory cytokines, MMP-3 and -13, and ADAMTS-4 and -5, while up-regulating COL-2 and Aggrecan levels in vitro, indicating its ability to alleviate inflammation and inhibit degeneration in rat nucleus pulpous cells.	[60]
PNIPAAm (N-isopropylacrylamide-based thermosensitive hydrogels)	SHP099	Promotes the expression of key proteins (collagen II and aggrecan) and reverses the degeneration of NP cells	SHP2 inhibited the expression of Sox9, collagen II, and aggrecan, leading to IVD degeneration. SHP099, a small-molecule inhibitor of SHP2, reversed the degeneration of nucleus pulposus (NP) cells in vitro. The Immunohistochemistry analysis showed that SHP2 was upregulated in degenerated IVDs, while cartilage-related genes were downregulated.	[61]
PEG polyethylene glycol (PEG) hydrogel	miRNA874	Regulates the synthesis/catabolism balance of ECM	Agomir874, a modified miRNA, can down-regulate the expression of matrix metalloproteinases (MMPs) in the nucleus pulposus (NP) The PEG-hydrogel is biocompatible and has little toxicity to human fibroblasts and rat NPCs, making it suitable for various tissue regeneration processes. PEG-hydrogel loaded with Agomir874 can be injected into the intervertebral space to regulate the synthesis ECM in the NP and improve tissue microenvironment regeneration, and its mechanical strength can be adjusted to match the condition of the intervertebral disk.	[62]
ZOGA (zinc-oxidized sodium alginate-gelatin)	Antagomir-204–3p	Inhibits the apoptosis of NPCs; maintains the metabolic balance of the ECM	The study developed a high-strength biohydrogel based on zinc-oxidized sodium alginate-gelatin (ZOGA) as a multifunctional platform for nucleic acid delivery, specifically antagomir-204-3p (AM), to ameliorate intervertebral disc degeneration (IVDD). Radiological and histological analyses showed that ZOGA restored the height of the IVD, retained moisture, and maintained tissue structure. Additionally, the study used enzyme catalysis and a combination of component units to construct the ZOGA biohydrogel, which exhibited stable porous and multichannel structures.	[63]
M2c-Exos@HA hyaluronic acid	Exosomes from M2c macrophages	Maintains the metabolic balance of ECM	M2c-Exoss derived from M2c macrophages have positive effects on the extracellular matrix metabolism in intervertebral disc degeneration (IVDD) through the miR-124/CILP/TGF-β regulatory axis; the M2c-Exos@HA hydrogel, loaded with M2c-Exoss, showed long-term alleviation of IVDD in rats. Cartilage intermediate layer protein (CILP) was identified as a key protein responsible for the rebalancing effects of M2c-Exoss on ECM metabolism in the nucleus pulposus (NP).	[64]
(CP-CS) chitosan poly(hydroxybutyrate-*co*-valerate) with chondroitin sulfate nanoparticles	Adipose-derived rat mesenchymal stem cells (ADMSCs)	Stimulates the proliferation and differentiation of stem cells	The composite hydrogel showed promising properties for nucleus pulposus tissue engineering, including similar swelling and viscoelastic properties to human nucleus pulposus and the ability to withstand different stress levels. It also supported chondrogenic differentiation of human mesenchymal stem cells.	[65]
CS/HA (chitosan (CS) and hyaluronic acid (HA) crosslinked with glycerol phosphate (GP) hydrogel)	ADSCs	Stimulates the proliferation and differentiation of stem cells	Salvianolic acid B (SalB) effectively reduced the apoptosis of bone marrow mesenchymal stem cells (BMSCs) in vitro by activating the JAK2-STAT3 pathway. The addition of SalB to 1% HAMA hydrogels improved the water retention and cell survival within the hydrogels. The HAMA + SalB + BMSCs groups are capable of extending cell morphology and maintaining normal cell shape, and had more pronounced delaying effect on the progression of disc degeneration compared to other treatment groups in the in vivo rat model.	[66]
CS/HA Hydrogels	Human adipose-derived stem cells (ADSCs))	Stimulates the proliferation and differentiation of stem cells	The hydrogel showed different swelling ratios based on the proportions of chitosan and hyaluronic acid, with higher ratios of hyaluronic acid resulting in increased swelling and exhibiting similar mechanical properties to native nucleus pulposus tissue. The hydrogel with encapsulated adipose-derived stem cells and kartogenin was released in a sustained manner, which promoted adipose-derived stem cell proliferation and nucleus pulposus differentiation. The hydrogel with a ratio of 5:3:2 showed the highest cell distribution and was comparable to fresh nucleus pulposus in terms of compressive modulus.	[67]
Gel-PEG-tyramine (gelatin/poly-(ethylene glycol)/tyramine)	Simvastatin	Promotes the production of ECM	Discs treated with 5 mg/mL of simvastatin in hydrogel or saline showed normal MRI indices through 8 weeks after treatment and lower histologic grades compared to saline only or hydrogel only, as well as the corresponding higher doses of 10 and 15 mg/mL. BMP-2 expression was upregulated at all doses of simvastatin in both saline and hydrogel carriers, with increased expression over time in the hydrogel plus 5 mg/mL of simvastatin group.	[68]
FibGen/CHS (genipin crosslinked fibrin/collagen type I)	Infliximab	Anti-inflammatory	The study demonstrated that genipin-crosslinked fibrin (FibGen) with collagen type I hollow spheres (CHS) can serve as a drug delivery carrier for the anti-TNFα drug, infliximab. FibGen loaded with higher concentrations of infliximab resulted in almost 6x greater drug release compared to lower concentrations. The released infliximab from FibGen remained bioactive and effectively inhibited the production of pro-inflammatory cytokines by human AF cells.	[69]
FBG-HA(Fibrin/hyaluronan)	BMP-2/7 heterodimer	Promotes the expression of type II collagen and the synthesis of glycosaminoglycans	Covalently incorporated BMP-27 can stimulate ACAN and COL2A1 expression and GAG synthesis of bovine NP cells cultured in FBG-HA hydrogel. In an IVD organ model, BMP-27 delivered into the nucleotomized region has the potential to promote the phenotype and synthesis of proteoglycan in the remaining NP, without causing fibroblastic or osteogenic induction. delivery of BMP-27 may be a promising therapeutic approach for NP regeneration.	[70]

**Table 5:** The recent approaches and their in vitro findings of hydrogel-based biomaterials for the Intervertebral Disc Degeneration.

Hydrogels	Major Findings	Reference
Oligo [poly (ethylene glycol) fumarate]/sodium methacrylate (OPF/SMA) hydrogel loaded with PLGA microspheres containing IL-4 (IL-4-PLGA) and kartogenin (KGN-PLGA)	The OPF/SMA showed good mechanical properties and low immunogenicity, promoting the sustained release of drugs. It induced macrophages to transition from the M1 phenotype to the M2 phenotype and exhibited a continuous anti-inflammatory effect. The scaffold also regulated the local inflammatory microenvironment and continuously repaired tissue in the nucleus pulposus, resulting in increased proportions of M2 macrophages, higher expression levels of type II collagen and aggrecan, and reduced levels of MMP13 expression. Additionally, continuous anti-inflammatory repair improved the immune microenvironment in the intervertebral disc and reduced the immunogenicity of the material.	[20]
Nano(2D-Mno2)-Hybrid Peptide Hydrogel (NHPH) loaded with 1D dynamic peptide nanofibrils and growth differentiation factor 5(GDF-5)	The NHPH facilitated the structural and functional recovery of the IVD after severe injuries by delivering pro-regenerative cytokines in a sustained manner, suppressing immune responses, and restoring the regenerative microenvironment of the ECM. The addition of growth differentiation factor 5 (GDF-5) in the NHPH further promoted ECM regeneration and improved tissue repair in the AF and NP regions of the IVD.	[117]
Gelatin methacrylate (GelMA) mixed with Curcumin encapsulated in solid lipid nanoparticles (SLNs)	The Cur-SLNs/GelMA hydrogel scaffold promotes the remission of IVD by regulating the stability of extracellular matrix metabolism in the nucleus pulposus and reducing the inflammatory response. In vitro and in vivo studies demonstrated that Cur-SLNs inhibited the expression of inflammatory factors TNF-α and IL-6, and regulated the recovery of Col II and aggrecan in an inflammatory environment.	[118]
BIOGEL (gelatin-tetrazine (GelTz) + gelatin-norbornene (GelNb))	The BIOGEL system exhibited biocompatibility, biodegradability, and cell adhesion properties, making it suitable for in situ crosslinking; furthermore, the IVD tissue architecture score, including AF morphology, NP cellularity, NP matrix, and the boundary between AF and NP, was improved when supplemented with TGFβ.	[119]
Chitosan/alginate hydrogel incorporated with allopurinol loaded into chitosan nanoparticles	Allopurinol-loaded hydrogels showed higher cell viability, metabolic activity, higher percentage of in vitro wound closure, and released significantly lower amounts of IL-6 compared to control groups. Cells cultured on allopurinol-loaded hydrogels were more resistant against oxidative stress. Animals treated with allopurinol-loaded hydrogel had significantly higher tissue expression levels of type I and type II collagen genes compared to other groups. The relative expression levels of type I collagen, type II collagen, nuclear factor κB (NF-κB), and glutathione peroxidase (GPx) genes in the allopurinol-loaded hydrogel group were 2.77 ± 0.2%, 2.86 ± 0.25%, 0.58 ± 0.03%, and 0.45 ± 0.02%, respectively.	[120]
Mel-MBG/SA	The Mel-MBG/SA hydrogel exhibited desirable physical and mechanical properties comparable to those of natural load bearing IVD, with compressive loading capabilities ranging from 0.75 to 2.75 MPa, and it provided sustained release of melatonin, which attenuated IL-1β-induced oxidative stress and reduced inflammation. Melatonin showed potential in increasing the expression of nucleus pulposus phenotype-associated genes, collagen II (COL2A1) and aggrecan, indicating its ability to promote matrix formation within the IVD.	[121]
Decellularization of notochordal cell -derived matrix (NCM) with poly (ethylene glycol) gel	The gels exhibited shear-thinning behaviour and were tuneable over a wide range of stiffnesses and swelled up to 150% of their initial wet weight and retained their swollen weight for 28 days. The gels with encapsulated decellularized NCM (dNCM) showed slow effusion of dNCM into the surrounding media, with soft gels losing more dNCM than stiff gels. The release of dNCM was higher in media mimicking the degenerate IVD environment. Loss of dNCM was highest within the first 2 days and then slowed down. Soft gels lost 75% of dNCM in healthy IVD-like conditions and all of the encapsulated dNCM in degenerate IVD-like conditions, while stiff gels leached only up to 25% of their dNCM cargo. The viability of mesenchymal stromal cells (MSCs) remained high after injection into the gel, with stiffer gels trending toward lower viability values.	[33]
Self-healing DNA hydrogel was synthesized as a carrier for SNAs coated with miR-5590	The DNA hydrogel exhibited injectable, self-healing, biodegradable, and superabsorbent properties, and resulted in the formation of a gene-hydrogel microenvironment, triggering autophagy in nucleus pulposus cells (NPCs) and suppressing apoptosis. In vitro and in vivo experiments showed that the miR-5590-SNA-loaded DNA hydrogel regulated the metabolic equilibrium within the extracellular matrix, and led to the gradual restoration of intervertebral space height in degenerated discs impeding the progression of IVDD.	[122]
Vanillin-based multifunctional GelMA microspheres	The functionalized microspheres effectively improved the release kinetics of TGFβ3, inhibited inflammatory responses, and promoted the secretion of extracellular matrix (ECM) in lipopolysaccharide-induced nucleus pulposus (NP) cells in vitro. In vivo, the functionalized microspheres alleviated inflammation and oxidative stress, preserved the water content of the intervertebral disc (IVD), maintained the disc height and intact structure, and promoted the regeneration of the IVD. High-throughput sequencing suggested that the therapeutic effects of the functionalized microspheres might be associated with the inhibition of the phosphatidylinositol 3-kinase (PI3K)-Akt signalling pathway.	[123]
Girard reagent T-modified oxidized dextran (OG) and adipic acid dihydrazide (ADH)-grafted catecholcoupled gelatin (GCA)	The gene-drug release from the hydrogel demonstrated excellent hemocompatibility and cytocompatibility with rat NP cells and could be sustained for more than 28 days, inhibiting the P65/NLRP3 signaling pathway, the secretion of inflammatory factors, and the degeneration of nucleus pulposus (NP) cells induced by lipopolysaccharide. In vivo experiments confirmed the efficient and prolonged gene silencing effect of the hydrogel, as well as its ability to regulate the inflammatory microenvironment and achieve successful IVD regeneration.	[124]

**Table 6:** In vivo Studies using different formulations of hydrogels.

Materials	Hydrogel Composition	Biofunction	Disease Model	Reference
Platelet-rich plasma and ferulic acid hydrogel compound	Platelet-rich plasma (PRP) and ferulic acid (FA)	Cytocompatible; active role in promoting ECM synthesis, strengthening, and repairing degenerated IVDs; anti-inflammatory and maintenance of disc height	Rat	[125]
Hyaluronic Acid Hydrogel	Hyaluronic Acid	Disc height was increased at weeks four and eight; significantly downregulated inflammatory response, specifically downregulation of IL-6, as well as a slightly reduced IL-1β and TNF-α response	Rabbit	[126]
HYADD^®^4-G	Hyaluronic Acid Derivative	Restored disc height to over 75% of the pre-punctured disc, improved water retention, and restored normal disc appearance, tissue organization, and cellularity; resulted in significantly lower intervertebral disc degeneration scores	Rabbit	[127]
Fucoidan-DexMA composite hydrogel scaffold	Dextran, glycidyl methacrylate, 4-dimethylaminopyridine	Increased the proliferation of nucleus pulpous cells (NPCs) whilst enhancing the synthesis of ECM on NPCs via the CAV1-YAP signaling pathway [128]. The scaffold was also seen as capable of promoting the M2 polarization of infiltrating macrophages and effectively alleviate the inflammatory microenvironment	Mouse	[128]
Polyurethane scaffold (F-PECUU) loaded with fucoidan	Biocompatible poly (ether carbonate urethane) urea (PECUU) nanofibrous scaffold loaded with fucoidan	Alleviated the inflammation and oxidative stress caused by lipopolysaccharide-prevented extracellular matrix (ECM) degradation in AF cells, while promoting ECM deposition to maintain the height, water content, and mechanical property of discs.	Mouse	[129]
Biodegradable Microspheres and Hydrogel Drug Delivery System of Tumor Necrosis Factor (TNF) Inhibitor and Growth Differentiation Factor 5 (GDF5)	Biodegradable microspheres containing Etanercept and GDF5	Biocompatible; able to deliver sustained and therapeutic dosages of ETN and GDF5; long-term anti-inflammatory and regenerative effect of the IVD	New Zealand White rabbits	[130]
Odex/Teleostean/CEC	Dextran, chitosan, and teleostean triple interpenetrating network hydrogel	Promote cell adhesion, migration, and proliferation; anti-inflammatory	Goat	[131]
GDH	Genipin-cross-linked decellularized nucleus pulposus hydrogel	Good biocompatibility, able to induce expression of NP related genes	Rat	[66]

## Abbreviations

IVD	Intervertebral Disc
NP	Nucleus Pulposus
AF	Annulus Fibrosus
CEP	Cartilaginous Endplates
ECM	Extracellular Matrix
MMP	Matrix Metalloproteinase
VEGF	Vascular Endothelial Growth Factor
NGF	Nerve Growth Factor
BDNF	Brain-Derived Neurotrophic Factor
PCL	Polypractalone
PVA	Polyvinyl alcohol
PVP	Polyvinylpyrrolidone
PEG	Poly(Ethylene Glycol)
PEGDA	Polyethylene Glycol Diacrylate
PEG-LM	Peptide-functionalized poly(ethylene glycol)—Laminin
(ASP-Lips)	Aspirin liposomes
GelMA	Gelatin Methacryloyl
PNIPAAm	Poly-Nisopropylacrylamide
ZOGA	Zinc-Oxidized Sodium Alginate-Gelatin
CP-CS	Chitosan Poly(hydroxybutyrate-co-valerate) with Chondroitin Sulfate nanoparticles
CS/HA—GP	Chitosan and Hyaluronic Acid crosslinked with Glycerol Phosphate
SalB	Salvianolic acid B
BMSCs	Bone Marrow Mesenchymal Stem Cells
ADMSCs	Adipose Derived Rat Mesenchymal Stem Cells
FBG-HA	Fibrin/Hyaluronan
OPF/SMA	Oligo [Poly (ethylene glycol) Fumarate]/Sodium Methacrylate
SLN	Solid Lipid Nanoparticle
OG	Girard reagent T-modified Oxidized Dextran
ADH	Adipic Acid Dihydrazide
GCA	Grafted Catecholcoupled Gelatin
NCM	Notochordal Cell derived Matrix
(dNCM)	Decellularized NCM
Mel-MBG/SA	Melatonin—mesoporous bioactive glass/sodium alginate
PECUU	Poly (Ether Carbonate Urethane) Urea
TNF	Tumor Necrosis Factor
GDF5	Growth Differentiation Factor 5
PRP	Platelet-Rich Plasma and
FA	Ferulic Acid
ETN	Etanercept
HAMA	Hyaluronic acid methacryloyl
BIOGEL	Bioorthogonal Hydrogel
GelTz	Gelatin-Tetrazine
GelNb	Gelatin-Norbornene
HYADD	Hyaluronic Acid Derivative
DexMA	Glycidyl methacrylate
